# The Fetal Effect of Maternal Caffeine Consumption During Pregnancy—A Review

**DOI:** 10.3390/biomedicines13020390

**Published:** 2025-02-06

**Authors:** Rajani Dube, Subhranshu Sekhar Kar, Shadha Nasser Mohammed Bahutair, Manjunatha Goud Bellary Kuruba, Shehla Shafi, Huma Zaidi, Heena Chaitanya Garg, Yumna Mushrmita Almas, Alweena Kidwai, Reem Ashraf Fathy Zalat, Omnia Elrasheid Babikir Sidahmed

**Affiliations:** 1 Department of Obstetrics and Gynecology, RAK College of Medical Sciences (RAKCOMS), RAK Medical & Health Sciences University (RAKMHSU), Ras Al-Khaimah P.O. Box 11172, United Arab Emirates; rajani.dube@rakmhsu.ac.ae (R.D.); shadha@rakmhsu.ac.ae (S.N.M.B.); yumna.19901077@rakmhsu.ac.ae (Y.M.A.); alweena.19901097@rakmhsu.ac.ae (A.K.); rim.19901091@rakmhsu.ac.ae (R.A.F.Z.); omnia.18901031@rakmhsu.ac.ae (O.E.B.S.); 2 Department of Pediatrics, RAK College of Medical Sciences (RAKCOMS), RAK Medical & Health Sciences University (RAKMHSU), Ras Al-Khaimah P.O. Box 11172, United Arab Emirates; 3 Department of Biochemistry, RAK College of Medical Sciences (RAKCOMS), RAK Medical & Health Sciences University (RAKMHSU), Ras Al-Khaimah P.O. Box 11172, United Arab Emirates; manjunatha@rakmhsu.ac.ae; 4 Department of Psychiatry, RAK College of Medical Sciences (RAKCOMS), RAK Medical & Health Sciences University (RAKMHSU), Ras Al-Khaimah P.O. Box 11172, United Arab Emirates; shehla@rakmhsu.ac.ae; 5 Department of General Education, RAK College of Medical Sciences (RAKCOMS), RAK Medical & Health Sciences University (RAKMHSU), Ras Al-Khaimah P.O. Box 11172, United Arab Emirates; huma@rakmhsu.ac.ae; 6 Department of Obstetrics and Gynecology, Al-Zahrawi Hospital, Ras Al-Khaimah P.O. Box 5973, United Arab Emirates; drhcgarg@gmail.com

**Keywords:** caffeine, pregnancy outcomes, fetal neurodevelopment, miscarriage, stillbirth, childhood obesity, leukemia

## Abstract

Caffeine is commonly used to excess by the general public, and most pregnant women drink caffeine on a daily basis, which can become a habit. Maternal caffeine intake during pregnancy is associated with severe gestational outcomes. Due to its lipophilic nature, caffeine can cross the blood–brain barrier, placental barrier, and even amniotic fluid. It can be found in substantive amounts in breast milk and semen. There has been a reported drop in neonatal anthropometric measurements with increased caffeine consumption in some cohort studies. This narrative review using literature titles and abstracts from the electronic databases of PubMed, Embase, and Scopus investigates the data linking maternal caffeine use to unfavorable pregnancy outcomes. It also evaluates the validity of the recommendations made by health professionals on caffeine consumption by mothers from the available literature. The results of our comprehensive literature search of case–control studies, cohort studies, randomized control trials, and meta-analyses, imply that caffeine use during pregnancy is linked to miscarriage, stillbirth, low birth weight, and babies that are small for gestational age. It was also found that there may be effects on the neurodevelopment of the child and links to obesity and acute leukemia. These effects can even be seen at doses well below the daily advised limit of 200 mg. The genetic variations in caffeine metabolism and epigenetic changes may play a role in the differential response to caffeine doses. It is crucial that women obtain solid, evidence-based guidance regarding the possible risks associated with caffeine.

## 1. Introduction

There is growing epidemiological evidence that maternal nutrition affects pregnancy outcomes, fetal growth and development, and the chance of developing diseases as an adult [1]. The optimal maternal diet should provide all essential nutrients. However, regardless of content, the maternal diet also contains pollutants and substances with pharmacological actions that may have negative effects. Caffeine is reported to be one of the most common drinks consumed by people worldwide, including pregnant women [2,3]. Caffeine consumption in adults is beneficial for certain cancers (such as prostate, melanoma, liver, and breast cancer), liver diseases (such as liver fibrosis and liver cirrhosis), type 2 diabetes, cardiovascular diseases (such as coronary heart disease and stroke), and neurological diseases (such as Parkinson’s disease and Alzheimer’s disease). However, caffeine use by pregnant women is linked with negative pregnancy outcomes [4,5]. It has also been linked to structural abnormalities and abnormal growth and development of the child [6,7,8]. Guidance from various countries advises women to limit their caffeine consumption to less than 200 mg daily (moderate) [9,10,11,12]. There is no local guidance for caffeine consumption in the UAE. Caffeine can be consumed in multiple forms, including coffee, espresso, tea, soda, dark chocolate, and breakfast cereals [3]. It is also reported that pregnant women consume more than the recommended amount throughout the world [13]. There is a need for comprehensive evidence on the effects of caffeine intake in pregnancy and the further growth and development of the resultant child. Furthermore, the possible mechanisms of these effects need to be understood.

## 2. Materials and Methods

Objective: This narrative review aims to provide a comprehensive overview of the current understanding of caffeine use in pregnancy and investigates the data linking maternal caffeine use to unfavorable pregnancy outcomes.

Inclusion Criteria:

1. Peer-reviewed articles focusing on caffeine use in pregnancy and pregnancy outcomes.
2. Studies and reviews published in English that contribute to the understanding of caffeine effects in offspring neonates and children.

Exclusion Criteria:

1. Non-peer-reviewed literature, such as editorials and opinion pieces.
2. Studies not focused on human neonatal populations, animal studies, or laboratory-based research.

Literature Search Strategy: A comprehensive literature search was conducted using the PubMed, Embase, and Scopus databases. The search incorporated MeSH terms and relevant keywords, including the following: “caffeine”, “pregnancy outcomes”, “fetal impacts”, “spontaneous abortion”, “miscarriage’, ‘small for gestational age’, “low birth weight”, “preterm delivery”, “stillbirth”, “childhood overweight”, “childhood leukemia”, and “fetal neurodevelopment”. The search employed Boolean operators (AND, OR) to ensure comprehensive coverage. Two reviewers independently screened the search results for relevance. They assessed the titles and abstracts, followed by a full-text evaluation of potentially eligible articles. Discrepancies were resolved through discussion or consultation with the third reviewer. Articles published up to 31 December 2023 were considered for review.

Data Extraction and Analysis: Key information was extracted from the selected articles, including author(s) and publication year, study type and design, population characteristics, key findings related to the fetal effect of maternal caffeine consumption during pregnancy, and future directions.

Synthesis of Information: The extracted data were synthesized through thematic analysis, resulting in categorized findings on interindividual differences in the reaction to caffeine, adverse pregnancy outcomes (miscarriage, stillbirth, small for gestational age, low birth weight), child growth (and neurodevelopment), and childhood diseases.

Limitations: This review acknowledges potential biases due to the selected literature and the subjective nature of narrative syntheses.

Ethical Considerations: No ethical approval was required for this review as it utilized published literature.

## 3. Results and Discussion

The review of the published literature yielded 54 observational studies, 1 randomized control trial, and 13 meta-analyses focusing on the effect of maternal caffeine use on pregnancy and childhood (Table 1). There are several cohorts and case–control studies from various regions of the world regarding the outcomes of miscarriage, birth weight, and stillbirth, but evidence on preterm birth is limited.

### 3.1. Caffeine Metabolism and Interindividual Differences in the Reaction to Caffeine

Human studies have demonstrated that the effects of caffeine on pregnancy outcomes vary greatly amongst individuals [27,28,29,30,31]. Over the past 20 years, there has been a major increase in scientific interest in the field of substantial interindividual phenotypic variation and the underlying mechanisms in complex characteristics and disorders. The idea that a person’s susceptibility to disease is a complicated readout of the interactions between their environment, genetic makeup, and epigenetic modifications during development is becoming more and more acknowledged [32,33]. The relative contributions of these several factors to interindividual variation and disease propensity vary from case to case, rely on particular situations, and occasionally exhibit notable synergism. Caffeine exerts its stimulant action through adenosine receptors. Both the sensitivity of adenosine receptors and the metabolism of caffeine contribute to individual variance in response [34]. Specifically, control of Cytochrome P4501A2 (CYP1A2), the enzyme that limits the rate of metabolism of caffeine, is a thoroughly researched instance. CYP1A2 and N-acetyltransferase 2 (NAT2) are key enzymes in the metabolism of caffeine. Glutathione S-transferase alpha1 (GSTA1) conjugates glutathione to aromatic amines and may also be active in the metabolism of caffeine. According to epidemiological research, women with higher CYP1A2 enzyme activity (rapid caffeine metabolism) are more likely to experience pregnancy issues when exposed to the same dosages of caffeine compared to women with lower CYP1A2 enzyme activity [27,28,29,30,31]. Human CYP1A2 mRNA levels show inter-individual variations of over 40 times, and the 3-demethylation of caffeine allows for the investigation of up to 60 times variation in the CYP1A2 enzyme’s in vivo activity [35]. Apart from the hepatic constitutive expression of CYP1A2, various extrinsic and intrinsic factors can control the activity of CYP1A2, including cigarette smoking, excessive coffee consumption, inhibition by oral contraceptives, and co-regulation by other liver-enriched transcription factors [36,37]. In addition to genetic variants, the regulation of CYP1A2 by environmental factors may also entail numerous layers of epigenetic mechanisms. Studies have also shown that a combination of the status and activity of these metabolites may play a key role in the varied outcomes in individuals [38]. This is an attractive topic for future research and could result in individualized precision medicine.

### 3.2. Pregnancy Outcomes and Caffeine Use

Everyday consumption of caffeine, a xanthine alkaloid, is widespread around the world, and it is largely found in coffee, tea, energy drinks, chocolates, and some soft beverages. Caffeine can cross through the placental barrier. The fetal liver is immature, and primary enzymes that render caffeine inactive are not expressed by the fetus. Thus, caffeine excretion is delayed in the fetus. Caffeine metabolites can accumulate in the fetal brain [39,40,41]. During pregnancy, the metabolism of caffeine is greatly reduced, especially after the first trimester [42]. As the pregnancy progresses, the half-life of caffeine increases from 2.5 to 4.5 h to about 15 h [43]. This is due to the decreased activity of liver enzymes responsible for caffeine metabolism (by one-third in the first trimester and by half in the second) [44]. Mothers’ caffeine absorption may also build up in oviductal or uterine fluid settings [45]; this can further affect the embryonic development of the fetus.

**Figure 1:**
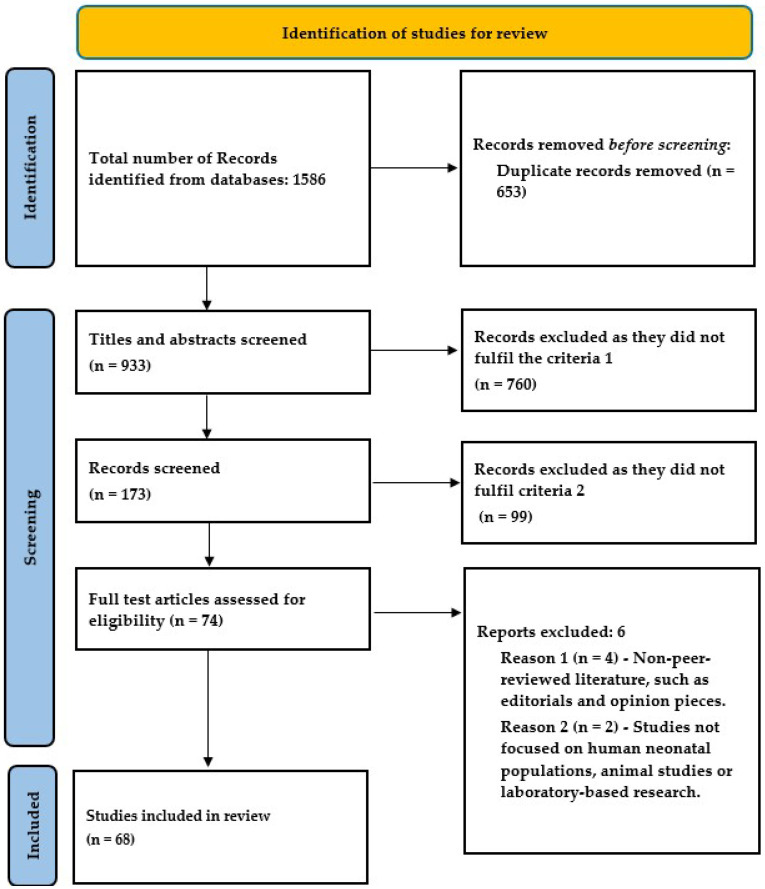
PRISMA (version 2020) flow diagram for study inclusion.

Furthermore, adrenaline, dopamine, and serotonin concentrations rise due to coffee consumption, which affects placental blood flow and the delivery of nutrients to the fetus through the placenta [20,46]. High coffee consumption during pregnancy may increase the fetus’s catecholamine levels, which may promote placental vasoconstriction [47] and raise the fetal heart rate, affecting the oxygenation of the fetus [48]. The symptoms of withdrawal like irritability and arrhythmias are also observed in newborns of mothers consuming caffeine during pregnancy, further substantiating the accumulation in the fetus in utero. Figure 1 summarizes the possible explanation of the effects of maternal caffeine intake on the fetus.

Up until recently, the majority of specialists recommended that pregnant women consume no more than 300 mg of caffeine each day [16]; however, recent recommendations of the European Food Safety Authority (EFSA) and the American Institute of Medicine have limited the amount to 200 mg/day [12]. The reason for this may be the inter-individual variations in genes and epigenetic modification.

### 3.3. Adverse Pregnancy Outcomes

**Figure 2:**
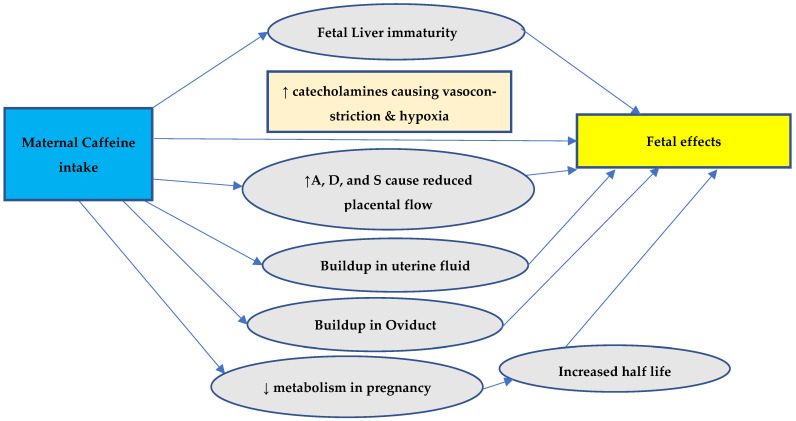
Possible mechanisms of adverse pregnancy outcomes. A = adenosine, D = dopamine, and S = serotonin.

A frequent element in the diet of pregnant women, caffeine is the most widely consumed psychoactive substance in the world [49]. Possible mechanisms of adverse pregnancy outcomes are shown in Figure 2. A putative link between caffeine use and perinatal morbidities is of concern since many women continue to drink coffee and other caffeinated drinks while pregnant. Reduced metabolism and increased half-life during pregnancy (15.08 vs. 4.71 h) cause buildup in the body, which crosses the placenta freely and causes fetal effects due to the inability to be metabolized by the fetal liver [50,51,52]. It is important that women receive advice about the negative pregnancy outcomes that can be caused by caffeine consumption [7].

There are a limited number of studies on structural anomalies associated with caffeine use. These studies have reported either no association or non-significant associations. However, there were considerable biases in both of these studies [13]. One of the unfavorable outcomes of pregnancy is fetal death—defined as the death of the fetus before complete evacuation from its mother—which is responsible for a significant fraction of perinatal mortality [53]. It can be separated into stillbirth and spontaneous abortion [22,54,55].

#### 3.3.1. Spontaneous Abortion/Miscarriage

Spontaneous abortion (SAB) is defined as the involuntary termination of a pregnancy leading to fetal death before 20 weeks of gestation [56].

It has been reported that coffee use alters the levels of endogenous hormones [57,58,59,60]. Caffeine was shown to be favorably correlated with sex hormone-binding globulin [59] and negatively correlated with levels of estradiol and progesterone during the luteal phase of the menstrual cycle [58,59,60]. Thus, it is plausible that hormonal alterations brought on by caffeine use might influence the chances of SAB [57].

Caffeine can cause vasoconstriction in the fetus via increased catecholamine output, resulting in decreased uterine and placental blood flow, leading to fetal hypoxia [46]. There were 30 observational studies and 4 meta-analyses reporting the outcomes of the risk of miscarriage (Table 2). These studies used questionnaire surveys as well as bio-marker levels as evidence of caffeine consumption. Most of the studies adjusted the confounders for SAB as maternal age, education status, economic status, ethnicity, pre-pregnancy body mass index, smoking, alcohol use, and exercise levels. All of the studies reported a significant risk of miscarriage with either pre-pregnancy consumption, during pregnancy, or both. Only one survey reported a suggestive association. This study also reported suspected recall bias [61]. However, the prospective studies were free from recall biases.

A comprehensive and up-to-date meta-analysis investigated the association between maternal coffee and caffeine and the risk of pregnancy loss and confirmed that coffee or caffeine consumption raises the risk of pregnancy loss [58]. In a prospective cohort study, it was shown that caffeine use during pregnancy was related to a higher risk of miscarriage and that there was a dose–response relationship, with the majority of the risk at 200 mg or more of caffeine per day. This observed impact was not affected by a number of possible confounders, such as pregnancy-related symptoms like nausea and vomiting or intolerance to coffee. There was an approximately 80% higher risk of miscarriage linked with caffeine use of 200 mg/day or more, even among women who did not alter their caffeine consumption habits while pregnant. Finally, caffeine intake from sources other than coffee revealed a comparable elevated risk of miscarriage, suggesting that the higher risk of miscarriage was caused by caffeine alone rather than other potential compounds in coffee. Independent of pregnancy-related symptoms, the data showed that consuming large amounts of coffee while pregnant increases the chance of miscarriage [62]. There is considerable agreement between all of the studies regarding the causation of SAB. However, the doses used as the reference were different, and multiple studies reported a non-linear association with the daily dose. A recent study also reported no threshold for safe intake [63]. This needs further research, keeping in mind the interindividual variation in caffeine metabolism.

**Table 2:** Caffeine intake and spontaneous abortion/miscarriage. OR: Odds Ratio.

Author and Year (Reference)	Country	Study Design	Events/Sample Size	Comments and Associate Factors
Purdue-Smithe et al. (2019) [63]	USA	Prospective Cohort		Pre-conceptional caffeine consumption is associated with increased risk. Biomarkers confirmed consumption. No safe threshold; miscarriage is not dependent on nausea or vomiting during pregnancy
Gaskin et al. (2018) [64]	USA	Prospective Cohort	2756/15,950 (17.2%)	Pre-pregnancy intake was associated with increased risk (not during early pregnancy). Factors like the age of the mother, BMI, smoking, alcohol, physical activity, history of infertility, race, and folate intake were considered and adjusted
Morales-Suárez-Varela et al. (2018) [65]	Denmark	Cohort	Risk: 1.22 (0.91–1.63)	Compared to no intake, >3 cups/day is associated with a higher risk of miscarriage. Age, parity, socio-economic status, physical exercise, alcohol, and BMI were also considered
Hahn et al. (2015) [66]	Denmark	Cohort	732/5132 (14.3%)	Age, physical activity, parity, BMI, education, smoking, and previous miscarriage
Agnesi et al. (2011) [67]	Italy	Case–Control	123/231 0.53	Maternal age and education. Focusses on the effect after community education and awareness
Stefanidou et al. (2011) [68]	Italy	Case–Control	52/312 (16.6%)	Maternal caffeine intake is associated with a three-fold increase in recurrent miscarriage per 100 mg daily intake. Not confounded by age, education, and tobacco intake
Greenwood et al. (2010) [17]	UK	Prospective Cohort	28/2635 (1.1%)	Increase in late miscarriage with consumption >300 mg in early pregnancy. Age, parity, smoking, and alcohol
Pollack et al. (2010) [69]	USA	Prospective Cohort	13/66 (19.6%)	Age, alcohol, smoking, and previous miscarriage
Zhang et al. (2010) [70]	China	Case–Control	326/726 (44.9%)	Age, alcohol, smoking, education, BMI, and previous history
Weng et al. (2008) [62]	USA	Prospective Cohort	172/1063 (16.2%)	Increased risk of miscarriage not confounded by age, race, education, income, previous miscarriage, alcohol smoking, pregnancy nausea, or vomiting
Savitz et al. (2008) [61]	USA	Cross-Sectional Cohort	258/2407 (10.7%)	Pre-pregnancy intake associated with increased risk. Age, ethnicity, education, alcohol, nausea, and vomiting were considered. Possibility of recall bias
Maconochie et al. (2007) [71]	UK	Case–Control	(603/6719)	No association between caffeine intake and miscarriage after adjusting for confounders. The primary aim was causes of miscarriage
Khoury et al. (2004) [72]	USA	Prospective Cohort	23/191 (12%)	Age, type-1 diabetes, previous SAB, nephropathy, retinopathy, glycemic control, and smoking were considered. Increased risk not confounded by smoking
Rasch et al., (2003) [73]	Denmark	Case–Control	OR: 2.21 (330/1498)	Daily intake of >375 mg is associated with increased risk. Age, parity, cigarette, and alcohol considered
Giannelli et al. (2003) [74]	UK	Case–Control	160/474 (33.7%)	Double the risk with consumption for >300 mg compared to ≤150 mg daily. The majority were non-smokers. Age and nausea were considered and adjusted
Tolstrup et al. (2003) [75]	Denmark	Case–Control	OR: 1.26 for 75–300 mg (303/1684)	Pre-pregnancy intake has a linear relation with miscarriage. Doses were <75, 75–300, 301–500, 501–900, and >900 mg. Age, smoking, and alcohol were considered as confounders
Wen et al. (2001) [76]	USA	Prospective Cohort	75/650 (11.5%)	Pre-pregnancy and early pregnancy intake. Increased risk not related to nausea and vomiting. Women with nausea had increased risk with intake ≥300 mg daily
Cnattingius et al. (2000) [77]	Sweden	Case–Control	562/1515 (37%)	Increased risk not confounded by age, history of SAB, alcohol, or pregnancy symptoms. Risk persists in non-smokers
Parazzini et al. (1998) [78]	Italy	Case–Control	782/1543 (50.6%)	OR: 1.2, 1.8, and 4.0 for 1, 2 to 3, and ≥4 cups per day, respectively. Age, education, previous miscarriages, alcohol, smoking, and severity of nausea were considered
Fenster et al. (1997) [79]	USA	Cohort	498/5144 (9.6%)	Pre-pregnancy and early pregnancy caffeine intake. Age, smoking, alcohol, obstetric history, and socio-economic status were adjusted
Dlugosz et al. (1996) [51]	USA	Cohort	135/2967 (4.6%)	Maternal age was also included as a risk factor
Al-Ansary et al. (1994) [80]	Saudi Arabia	Case–Control	226/452	Increased risk in caffeine intake >150 mg daily. Primary inquiry into causes of miscarriage
Dominguez- Rojas et al. (1994) [81]	Spain	Cohort	169/691 (24%)	Age and previous miscarriage
Infante-Rivard et al. (1993) [82]	Canada	Case–Control	OR: 2.62 (>321 mg daily during pregnancy) (331/1324)	Increased risk for consumption pre-pregnancy (OR: 1.85) and during pregnancy. Age, education, smoking, alcohol, and uterine malformations were confounders and adjusted. ORs increased by a factor of 1.22 for each 100 mg daily intake
Mills et al. (1993) [83]	USA	Cohort	59/423 (13.9%)	Smoking, alcohol intake, age, parity, education, and previous miscarriage were included as risk factors
Armstrong et al. (1992) [84]	Canada	Cohort	7760/35,848 (21.6%)	Age, education, and ethnicity were the considered risk factors
Parazzini et al. (1991) [85]	Italy	Case–Control	78/212 (36%)	Maternal age
Fenster et al. (1991) [86]	USA	Case–Control	OR: 1.22 (607/1891)	Dose dependent. Confounders were adjusted. Heavy consumption (>300 mg daily) with nausea doubled the risk (OR: 2.1)
Wilcox et al. (1990) [87]	USA	Cohort	43/171 (25%)	The association between miscarriage and risk factors was explored. Age, pregnancy history, weight, education, prenatal DES exposure, smoking, alcohol, and marijuana were other variables
Axelsson et al. (1989) [88]	Sweden	Cohort	126/1242 (10.1%)	Age, occupation, and smoking were other risk factors

#### 3.3.2. Stillbirth

For the birth of a fetus without any sign of life after 20 weeks of pregnancy or when it weighs 14 oz, the term “stillbirth” is used. It can be due to intrauterine demise in pregnancy or during childbirth [56]. There were eight observational studies and two meta-analyses focusing on the outcome of stillbirth or late fetal demise (Table 3).

Coffee consumption during pregnancy is linked to a higher risk of stillbirth [89]. In several ways, caffeine may raise the risk of late fetal mortality. One mechanism by which coffee may cause fetal hypoxia and vasoconstriction in the uteroplacental circulation is by increasing the release of catecholamines from the renal medulla [47,90]. Another possibility is that caffeine may directly affect a developing fetus’s circulatory system, causing tachycardia and other arrhythmias [48]. Compared to pregnant women who drank no coffee, the rate of stillbirth dropped in those who drank one to three cups per day, climbed slightly in those who drank four to seven cups per day, and more than doubled in those who drank eight or more cups per day. These findings seem to point to an impact threshold of four to seven cups per day [89]. Women should be made aware that caffeine use during pregnancy increases the chance of stillbirth, especially at doses higher than those advised by the World Health Organization (WHO) (>300 mg/day) [91]. These studies suggest a definite association of stillbirth with a higher intake of caffeine than the recommended amount. One study explored the gene pleomorphism for caffeine metabolism and reported a combination of the slow oxidizer status of CYP1A2, the slow acetylator status of NAT2, and the low activity of GSTA1 as being significantly associated with stillbirth [38].

**Table 3:** Caffeine and stillbirth. aOR: adjusted Odds Ratio. OR: Overall Risk. RR: Relative Risk.

Author and Year [Reference]	Country	Study Design	Events/Sample Size	Remarks and Associate Factors
Heazell et al. (2021) [91]	UK	Case–Control	aOR: 1.27 (1.14–1.43) for each 100 mg daily increase	290/1019 (28.4%) of stillbirth The attributable risk for stillbirth associated with >300 mg of caffeine/day was 7.4% Age, BMI, smoking, ethnicity, education, parity, and dietary supplements
Morales- Suárez-Varela et al. (2018) [65]	Denmark	Cohort	1178/90,086 (1.3%) Risk: 1.05 (0.62–1.77)	Compared to no intake, >3 cups/Day is associated with higher risk. Age, parity, socio-economic status, physical exercise, alcohol, and BMI were also considered
Gaskin et al. (2018) [64]	USA	Prospective Cohort	1.24 (0.57–2.69) ≥4 servings compared to never	There is a higher but non-significant risk of stillbirth with pre-pregnancy intake of >400 mg daily. Factors like the age of the mother, BMI, smoking, alcohol, physical activity, history of infertility, race, and folate intake were considered and adjusted
Greenwood et al. (2010) [17]	UK	Prospective Cohort	28/2635 (1.1%)	Increase in adverse outcomes with consumption >300 mg in early pregnancy. Compared to those consuming <100 mg/day, 2.2 for 100–199 mg/day, 1.7 for 200–299 mg/day, and 5.1 for >300 mg/day
Matijasevich et al. (2006) [92]	Uruguay	Case–Control	OR: 2.33 (382/1174)	Fetal death significantly more common with ≥300 mg/day. Other factors were education, previous miscarriage, pregnancy symptoms, and regular prenatal care
Bech et al. (2006) [38]	Denmark	Case–Control	RR: 2 (142/299)	Increased risk of stillbirth with a combination of slow metabolism genotypes of caffeine metabolism
Bech et al. (2005) [93]	Denmark	Prospective Cohort	1102/88,482 (1.2%)	The risk increased linearly with increased daily intake. Risk: 1.03, 1.33, and 1.59 with 1, 2–3, and >4 cups daily consumption of coffee, respectively. Age, parity, smoking, BMI, and alcohol intake were adjusted as confounders
Wisborg et al. (2003) [89]	Denmark	Prospective Cohort	82/18,478 (0.4%)	Caffeine intake during the first trimester is associated with stillbirth. Smoking, alcohol, parity, age, BMI, and education were other factors included

#### 3.3.3. Low Birth Weight (LBW)/Small for Gestational Age (SGA)

Low birth weight is defined as a birth weight of less than the 10th centile (−2 standard deviations from the mean). Small for gestational age on the other hand takes into account the gestational age and can represent a constitutionally small fetus or intrauterine growth restriction. Various factors can affect fetal growth in utero, including genetic, environmental, and metabolic, as well as infectious agents [55,94,95,96]. There were seventeen studies and seven meta-analyses that investigated the effect of maternal caffeine intake on the risk of LBW or SGA (Table 4).

The evidence is consistent with a risk of LBW or SGA in women with higher intake of caffeine prior to pregnancy and in early pregnancy. The only randomized trial demonstrated that moderate reduction of intake has no effect on birth weight or length of gestation [93]. A prospective cohort study in Finland included 2007 women in the first and 4362 in the third trimester of pregnancy for a survey on the intake of caffeine during pregnancy. They reported that more than 30% of women exceeded the recommended daily intake during pregnancy. The adjusted odds ratio (aOR) of SGA was 1.87 (1.16–3.02) in women with moderate (51–200 mg/day) and aOR 1.51 (1.08–2.10) in women with high (>200 mg/day) caffeine intake during the first trimester. Caffeine intake in the third trimester of pregnancy was not associated with SGA [97]. This demonstrates that even the recommended daily intake can cause adverse fetal effects. This may be explained by the polymorphisms of the genes that encode enzymes responsible for caffeine metabolism.

#### 3.3.4. Preterm Birth

Preterm birth is defined as the commencement of spontaneous labor before 37 weeks of gestation [16]. Preterm birth increases the risk of neurodevelopmental, pulmonary, and gastrointestinal problems, and, later on, childhood obesity [111,112,113,114,115], as well as hypertension [115] and reduced insulin levels later in life [116]. It is a major cause of infant mortality [38]. One of the prenatal exposures examined for a potential relation to preterm birth is pregnant women’s caffeine use [16].

A meta-analysis of cohort and case–control studies found no evidence of a relationship between caffeine use during pregnancy and the risk of preterm birth. This study, however, was unable to draw any conclusions about caffeine intakes over 300–400 mg/d because the majority of studies used this as their upper limit [16]. Kobayashi et al. (2019) investigated dose-dependent associations between prenatal caffeine consumption and adverse birth outcomes, including preterm birth, using data from the Japan Environment and Children’s Study. The study found that maternal caffeine intake was significantly associated with an increased risk of preterm birth, small for gestational age (SGA), and reduced birth weight. A key finding was that even moderate caffeine consumption (100–200 mg per day) elevated the risk of preterm birth compared to low or no caffeine intake. The study emphasized the need for culturally specific recommendations on caffeine consumption during pregnancy, given differences in dietary habits and caffeine sources [100]. Okubo et al. (2015) focused on maternal caffeine intake from Japanese and Chinese teas, a prevalent source of caffeine in the Japanese population. Their findings indicated a significant association between higher caffeine intake and preterm birth. The study highlighted that tea-based caffeine sources had similar effects to coffee, suggesting that total caffeine intake—regardless of source—is a critical factor in influencing pregnancy outcomes. The authors posited that caffeine’s potential to increase circulating catecholamines and stress hormones could contribute to uterine contractions and preterm labor [104]. Most studies found no significant link between caffeine use during pregnancy and the risk of preterm delivery [93,117]. A Cochrane review also did not find any evidence of benefit from the reduction of caffeine intake in pregnancy on preterm delivery. However, it only included two studies with low-quality evidence [118].

### 3.4. Effect of Maternal Caffeine Use on Childhood Development and Disease

#### 3.4.1. Neurodevelopment

The placental barrier is easily crossed by caffeine, a potent neuromodulator that is extensively utilized, but little is known about the long-term effects of gestational caffeine exposure (GCE) on neurodevelopment. In a study, magnetic resonance imaging (MRI) data were used to examine the structural results of the brain in 27 key fiber pathways as a function of GCE. The MRI data were obtained from a very large sample of 9157 children, aged 9–10 years, as part of the Adolescent Brain and Cognitive Developments study [58,119]. Using mixed effects binomial regression, significant correlations between GCE and fractional anisotropy (FA) values in the left hemisphere’s inferior fronto-occipito fasciculus and corticospinal tract were found. Further investigations were conducted to study the interaction between these fiber tracts, GCE, cognitive measures (working memory, task efficiency), and psychopathology measures (externalization, internalization, somatization, and neurodevelopment) [119]. GCE was associated with worse results on all psychiatric tests but had no influence on cognitive assessments. Higher FA levels in both fiber tracts were linked to fewer neurodevelopmental issues. Both cognitive tasks resulted in better performance. These findings imply that GCE can contribute to future neurodevelopmental difficulties, which occur in part due to changes in the architecture of key proteins. These findings imply that the current recommendations for reducing caffeine use during pregnancy might need modification [119,120]. A recent study from Japan involving 1199 mother–child pairs showed that higher maternal caffeine consumption during pregnancy was independently associated with a reduced risk of peer problems in the children and was not related to the risk of emotional problems, conduct problems, or hyperactivity in the children [121].

#### 3.4.2. Childhood Obesity/Overweight

Childhood overweight and obesity have become significant public health challenges worldwide, with their origins often tracing back to prenatal and early-life exposure. Maternal caffeine intake during pregnancy has been studied as a potential factor influencing the offspring’s growth patterns, body mass index (BMI), and adiposity. Voerman et al. (2016) conducted a prospective cohort study examining maternal caffeine intake during pregnancy in relation to early growth and body fat distribution in children of school age. It was reported that moderate and high maternal caffeine intake was associated with greater total body fat and abdominal fat in offspring [103]. In another study, the prevalence of overweight rose by 5% at three years, 6% at five years, and 3% at eight years when prenatal caffeine intake increased from low to extremely high. Children born to average-, high-, and very-high-caffeine consumers had 1.05, 1.17, and 1.44 greater adjusted odds of being overweight at three years of age, respectively, than children born to low caffeine consumers. Similar ORs were discovered at the age of five years; however, at eight years of age, the risk was substantial only for the highest caffeine consumption category. The exclusion of pregnant smokers or SGA neonates had no effect on the outcomes [122]. A similar linear relationship between maternal caffeine consumption as a continuous variable and the risk of being overweight at ages three and five, with a greater OR at age three than at age five was observed in another study [123,124].

Yet another study demonstrated that even intake of less than 300 mg/day was strongly linked to an elevated risk of excess infant development and obesity, even after omitting very high users and using non-drinkers as the reference group. Finally, when growth data from actual measures were used to investigate the link between maternal coffee use and overweight at different age stages, comparable trends and relationships were seen [125]. It was also reported that at any amount above 50 mg/day, caffeine was linked to increased BMI from one month to eight years but was associated with faster height gain only up to three months of age [126]. Papadopoulou et al. (2018) utilized data from a large Norwegian cohort to investigate the relationship between maternal caffeine intake and childhood growth trajectories. Their findings corroborated previous studies about the association, notably, the associations across various caffeine sources, including coffee, tea, and soft drinks, emphasizing that total caffeine intake—rather than specific sources—is critical to consider [122].

Chen et al. (2019) examined parental and grandparental caffeine intake in relation to childhood obesity. Their analysis indicated that only maternal caffeine intake was significantly associated with higher childhood BMI and adiposity, proposing a direct influence on the intrauterine environment [22]. When maternal serum paraxanthine (a primary caffeine metabolite) levels were analyzed in association with offspring BMI at ages 4 and 7 years, a weak positive association was observed [127]. Thus, the potential biological mechanisms behind the association between maternal caffeine consumption and childhood obesity are believed to be fetal programming, impaired placental function, epigenetic modifications, and rapid infant growth.

#### 3.4.3. Childhood Leukemia

Childhood acute leukemia (AL), including acute lymphoblastic leukemia (ALL) and acute myeloid leukemia (AML), represents the most common malignancy in children. While its etiology remains multifactorial, maternal exposures during pregnancy, including caffeine consumption, have been investigated for their potential role in influencing leukemia risk. Menegaux et al. (2007) conducted a case–control study in France examining maternal coffee and alcohol consumption, alongside parental smoking, as risk factors for childhood leukemia. Their findings suggested a potential association between high maternal coffee intake (defined as three or more cups per day) and an increased risk of AL [128]. The study noted that caffeine may influence fetal development through its effects on DNA replication and repair, potentially leading to oncogenic mutations. However, the authors emphasized the need for further research to corroborate these findings and explore underlying mechanisms. Building on prior research, Bonaventure et al. (2013) explored the interplay between maternal caffeine consumption and genetic predispositions in a case–control study. They observed an increased risk of ALL in children born to mothers with higher coffee intake during pregnancy, particularly among those with specific metabolic polymorphisms affecting caffeine metabolism. These findings highlight the potential role of gene-environment interactions in modulating leukemia risk. The study underscored the importance of considering individual metabolic differences when assessing the impact of maternal caffeine consumption [129]. Milne et al. (2011) reported an elevated risk of ALL associated with higher maternal coffee intake, with a dose–response relationship. Tea consumption, however, did not demonstrate a significant association. This study reinforced the hypothesis that caffeine exposure may disrupt fetal hematopoiesis or immune system development, potentially increasing leukemia susceptibility [64]. In a subsequent pooled analysis involving data from multiple international studies, Milne et al. (2018) further confirmed a modest but statistically significant increase in ALL risk with higher coffee consumption during pregnancy [23,130]. Importantly, this study addressed potential confounders, such as socioeconomic status and parental smoking, to strengthen the reliability of its findings. The authors proposed that caffeine’s potential to induce oxidative stress and impair DNA integrity might contribute to leukemogenesis. Focusing specifically on AML, Karalexi et al. (2019) utilized data from the Childhood Leukemia International Consortium to investigate maternal coffee and tea consumption during pregnancy. Unlike studies on ALL, they did not identify a significant association between caffeine intake and AML risk. The authors suggested that differences in leukemia subtypes and their underlying pathophysiology might explain the lack of correlation. They also noted the limited sample size for AML cases, which could affect the statistical power of the analysis [131]. The ESTELLE study by Orsi et al. (2015) examined maternal caffeine consumption in relation to childhood AL within a broader context of parental lifestyle factors. While the study did not find a definitive association between moderate coffee or tea intake and AL risk, it suggested that very high levels of caffeine consumption (e.g., five or more cups per day) might contribute to a slight increase in risk. The study also highlighted the complexity of disentangling caffeine’s effects from other maternal and environmental exposures [132].

The potential mechanisms linking maternal caffeine consumption to childhood leukemia involve caffeine’s known biological effects on cell proliferation, DNA repair, and oxidative stress. High levels of caffeine intake may disrupt normal fetal development by inducing DNA damage or impairing immune system maturation, which are critical processes in preventing malignancies like leukemia. Additionally, genetic polymorphisms affecting caffeine metabolism may modulate individual susceptibility. For example, slow caffeine metabolizers may experience prolonged exposure to caffeine’s metabolites, potentially amplifying its adverse effects on fetal development. Studies such as those by Bonaventure et al. (2013) highlight the importance of considering these genetic factors in understanding the risk associated with maternal caffeine intake [130].

Despite the suggestive evidence, several limitations warrant caution in interpreting these findings. First, most studies rely on self-reported dietary intake, which is subject to recall bias and inaccuracies. Second, confounding factors such as maternal diet, stress levels, and other lifestyle behaviors may influence the observed associations. While many studies attempt to adjust for these variables, residual confounding cannot be ruled out. Moreover, the heterogeneity of findings across studies underscores the complexity of this relationship. For instance, while some studies identify a dose–response relationship, others find no significant association, particularly for AML. Differences in study populations, sample sizes, and exposure assessment methods may contribute to these discrepancies. The relationship between maternal caffeine consumption during pregnancy and childhood acute leukemia remains an area of active investigation. Evidence suggests a modest association, particularly for ALL, with higher maternal coffee intake. However, the findings are not uniform, and further research is needed to elucidate the underlying mechanisms, account for genetic variability, and refine exposure assessment methods. Pregnant individuals may benefit from adhering to current recommendations to limit caffeine intake as a precautionary measure while future studies continue to explore this important public health question.

## 4. Conclusions

It is well acknowledged that prolonged chemical exposure during pregnancy should be taken seriously. Caution is important when the chemical under investigation is caffeine, which is a highly addictive and non-nutritional drug that is taken by almost everyone. There is a strong body of cumulative data linking caffeine usage by mothers to a variety of unfavorable pregnancy outcomes. Significant dose–response relationships indicative of causation are frequently reported in observational studies and meta-analyses, as well as numerous reports of no intake threshold below which associations are absent. As a result, the data available now do not support medical advice that suggests “moderate” caffeine use during pregnancy is safe. Contrarily, a growing body of scientific research suggests that women who are pregnant or considering becoming pregnant should steer clear of caffeine. Studies have produced experimental and epidemiological data indicating that examining the processes of caffeine response and genetic variations in more detail might open up new possibilities for precision medicine. Creating a quick and easy way to assess a person’s sensitivity to caffeine would help females monitor their own health as well as that of the fetus during pregnancy.

## Figures and Tables

**Table 1:** Meta-analyses.

Author and Year (Reference)	Daily Intake of Caffeine	Adverse Outcomes	Risk of Adverse Outcomes (CI)
Fernandes et al., 1998 [14]	<150 mg vs. ≥150 mg	Miscarriage	1.36 (1.29 to 1.45)
		Low birth weight (LBW)/Small for gestational age (SGA)	1.51 (1.39 to 1.63)
Santos et al., 1998 [15]	Low vs. High	LBW/SGA	1.29 (1.18 to 1.41) none vs. any 1.24 (1.05 to 1.43) low vs. high
		Preterm birth	Indeterminate none/low vs. high
Maslova et al., 2010 [16]	Lowest vs. Highest	Preterm birth	No important association
Greenwood et al., 2014 [17]	Every 100 mg	Miscarriage	1.14 (1.10 to 1.19)
		Preterm birth	1.2 (−1.02 to 1.06)
		SGA	1.10 (1.06 to 1.14)
		LBW	1.07 (1.01 to 1.12)
		Stillbirth	1.19 (1.05 to 1.35)
Cheng et al., 2014 [18]	None/low vs. highest	Childhood leukemia	1.72 (1.32 to 2.16)
Li et al., 2015 [19]	<150 mg vs. ≥150 mg	Miscarriage	1.32 (1.24 to 1.40)
Rhee et al., 2015 [20]	Lowest vs. highest	LBW/SGA	1.38 (1.10 to 1.73)
Thomopoulos et al., 2015 [21]	Lowest vs. highest	Childhood leukemia	1.57 (1.16 to 2.11)
Chen et al., 2016 [22]	Every 100 mg	Miscarriage	1.07 (1.03 to 1.12)
		Stillbirth	1.09 (1.02 to 1.16)
		Preterm birth	1.13 (1.06 to 1.21)
Milne et al., 2018 [23]	None vs. 3+ cups	Childhood leukemia	1.67 (1.20 to 2.32)
Jin et al., 2021 [24]	Every 100 mg	LBW	1.07 (1.02 to 1.11)
		Childhood obesity/overweight	1.31 (1.11 to 1.55)
Soltani et al., 2021 [25]	Every 100 mg	LBW	1.12 (1.03 to 1.22)
Askari et al., 2023 [26]	Every 100 mg	LBW	1.28 (1.16 to 1.41)
		Preterm birth	1.04 (0.95 to 1.14) No increased risk

**Table 4:** Caffeine and small for GA/LBW. aOR: adjusted Odds Ratio.

Author and Year [Reference]	Country	Study Design	Risk and Sample Size	Comments and Associate Factors
Kukkonen et al. (2024) [97]	Finland	Prospective cohort	aOR: 1.87 (moderate) aOR: 1.51 (high) (7944)	Caffeine intake of >50 mg daily in the first trimester was associated with SGA. Associated factors including age, BMI, smoking, and energy intake were taken as confounders and adjusted
Gleason et al. (2021) [98]	USA	Prospective cohort	β = −84.3 g (−145.9 to −22.62) (788)	Compared the anthropometric parameters with caffeine levels (≤28 ng/mL compared to >659 ng/mL)
Modzelewska et al. (2019) [99]	Norway	Cohort	1.16 (1.10 to 1.23) (67,569)	Being SGA increased neonatal morbidity and mortality. However, caffeine exposure per se was not associated with neonatal morbidity/mortality
Kobayashi et al. (2019) [100]	Japan	Prospective cohort	1.18 (1.10 to 1.27) (94,876)	Compared the intake of <86.4 mg/day and >205 mg/day. Also increased risk of preterm birth in the second trimester (RR: 1.94 (1.12 to 3.37))
Chen et al. (2018) [101]	Ireland	Prospective cohort	1.47 (1.14, 1.90)—Caffeine 3.10 (1.08−8.89)—Coffee (941)	There were similar findings for the intake of tea. Birth weight decrease of 71.9 g per 100 mg daily increase in caffeine intake
van der Hoeven et al. (2017) [102]	The Netherlands	Prospective Cohort	2 days (0.07–3.93) (936)	Caffeine >300 mg daily intake was associated with increased gestational age compared to consumption <100 mg. No associations between coffee consumption and birth weight. Smoking and maternal age were adjusted
Voerman et al. (2016) [103]	The Netherlands	Prospective Cohort	Birth weight (−55 g) (7857)	Compared to <180 mg, ≥540 mg daily intake was significantly associated with a decrease in birth weight (*p* < 0.001)
Okubo et al. (2015) [104]	Japan	Prospective Cohort	(858)	No association between total caffeine intake and the risk of LBW or SGA
Bech et al. (2015) [105]	Denmark	Prospective Cohort	aOR: 1.51 (1.21–1.88) (71,000)	Women drinking >8 cups/day have a high risk of LBW. Linear relationship of 9 g/cup/day
Hoyt et al. (2014) [2]	USA	Case–control	aOR: 1.3–2.1 (7943)	LBW was seen in 8.2%. Associated with LBW with intake of >300 mg per day
Sengpiel et al. (2013) [106]	Norway	Cohort	1.62–1.27 (59,123)	Risk is dose-dependent with maximum doses >200 mg. Risk varies according to the criteria used for SGA diagnosis. No increased risk for preterm birth
Bakker et al. (2010) [107]	The Netherlands	Prospective Cohort	(7346)	≥6 cups daily intake of coffee associated with SGA but not LBW.
CARE study group (2008) [27]	UK	Prospective Cohort	OR: 1.4 (1.0–2.0) for >300 mg. (2635)	Pre-pregnancy and pregnancy intake of caffeine is associated with growth restriction. OR: 1.2 (0.9 to 1.6) for 100–199 mg/day and 1.5 (1.1 to 2.1) for 200–299 mg/day intake of caffeine
Bech et al. (2007) [93]	Denmark	Randomized Control Trial	(1207)	There was no difference in birth weight and gestational age with a moderate reduction in the second half of pregnancy. Adjustment for length of gestation, parity, BMI, and smoking
Bracken et al. (2003) [108]	USA	Prospective Cohort	28 g per 100 mg daily (2291)	Urinary metabolites were also assessed. No increase in growth restriction, LBW, or preterm delivery
Clausson et al. (2002) [109]	Sweden	Prospective Cohort	(953)	No association between maternal caffeine intake and LBW or SGA
Klebanoff et al. (2002) [110]	USA	Prospective Cohort	(2515)	Significant risk of SGA only in smokers who consume caffeine. Adjusted for maternal age, pre-pregnant weight, education, parity, and ethnicity

## Data Availability

No new data were created or analyzed in this study. Data sharing is not applicable to this article.
